# Improving the adaptability of WHO evidence-informed guidelines for nutrition actions: results of a mixed methods evaluation

**DOI:** 10.1186/s13012-017-0571-2

**Published:** 2017-03-21

**Authors:** Maria Cecilia Dedios, Alexo Esperato, Luz Maria De-Regil, Juan Pablo Peña-Rosas, Susan L. Norris

**Affiliations:** 1 Independent consultant, Bogotá, Colombia; 2 Independent consultant, Washington DC, USA; 3 Evidence and Program Guidance, Department of Nutrition for Health and Development, World Health Organization, Geneva, Switzerland; 4 Guideline Review Committee Secretariat, World Health Organization, Geneva, Switzerland

**Keywords:** Guidelines, Nutrition, WHO, Adaptability, Implementability, Methodological quality, Mixed methods

## Abstract

**Background:**

Over the past decade, the World Health Organization (WHO) has implemented a standardized, evidence-informed guideline development process to assure technically sound and policy-relevant guidelines. This study is an independent evaluation of the *adaptability* of the guidelines produced by the Evidence and Programme Guidance unit, at the Department of Nutrition for Health and Development (NHD). The study systematizes the lessons learned by the NHD group at WHO.

**Methods:**

We used a mixed methods approach to determine the adaptability of the nutrition guidelines. Adaptability was defined as having two components; methodological quality and implementability of guidelines. Additionally, we gathered recommendations to improve future guideline development in nutrition actions for health and development. Data sources for this evaluation were official documentation and feedback (both qualitative and quantitative) from key stakeholders involved in the development of nutrition guidelines. The qualitative data was collected through a desk review and two waves of semi-structured interviews (*n* = 12) and was analyzed through axial coding. Guideline adaptability was assessed quantitatively using two standardized instruments completed by key stakeholders. The *Appraisal Guideline for Research and Evaluation* questionnaire, version II was used to assess guideline quality (*n* = 6), while implementability was assessed with the electronic version of the *GuideLine Implementability Appraisal* (*n* = 7).

**Results:**

The nutrition evidence-informed guideline development process has several strengths, among them are the appropriate management of conflicts of interest of guideline developers and the systematic use of high-quality evidence to inform the recommendations. These features contribute to increase the methodological quality of the guidelines. The key areas for improvement are the limited implementability of the recommendations, the lack of explicit and precise implementation advice in the guidelines and challenges related to collaborative work within interdisciplinary groups.

**Conclusions:**

Overall, our study found that the nutrition evidence-informed guidelines are of good methodological quality but that the implementability requires improvement. The recommendations to improve guideline adaptability address the guideline content, the dynamics shaping interdisciplinary work, and actions for implementation feasibility. As WHO relies heavily on a standardized procedure to develop guidelines, the lessons learned may be applicable to guideline development across the organization and to other groups developing guidelines.

## Background

Over the past decade, the World Health Organization (WHO) has implemented a standardized process to assure technically sound and country-relevant guidelines. In the past, WHO’s guideline development process relied heavily on expert opinion, an approach that raised criticism from the scientific community. In 2005, the organization launched a comprehensive undertaking to guarantee guideline quality. This included devising a new process to ensure that WHO recommendations were informed by the best available evidence [1]. This process now entails a thorough review of the available evidence through comprehensive systematic reviews of the effects of interventions using state-of-the-art methods for evidence retrieval, assessment, and synthesis that contribute to the rigor of the overall process [2, 3]. To this end, the WHO Handbook for Guideline Development [4, 5] outlines nine steps necessary for guideline development. Since January 2009, all WHO technical units have adhered to the methods and processes laid out in the Handbook when developing guidelines.

The Evidence and Programme Guidance, a unit with normative functions at the Department of Nutrition for Health and Development (NHD) at WHO, has followed the Handbook closely to develop over 20 guidelines on nutrition-specific or nutrition-sensitive interventions in collaboration with several internal partners. The group has taken some additional measures to ensure a steady increase in the methodological rigor of the guidelines. However, guidelines need not only to provide robust, reliable, and independent advise but also advise that is feasible and easily implemented in the real world [6]. While this is a hard to reach equilibrium, the NHD group is constantly seeking to incorporate these considerations in the guidance presented to users of these documents.

Innovations have been funneled by the group’s rigorous approach to guideline development, entailing the continuous monitoring, evaluation, and revision of the process. The present article is the product of an independent evaluation of the nutrition evidence-informed guideline development approach and systematizes the lessons learned by the NHD group at WHO. The results of the evaluation should further help to ensure that the guidelines meet their purpose and are adapted by Member States and partners.

## Methods

The objective of the evaluation was to assess the guideline-making process implemented by the NHD group at WHO. In order to do this, we sought to determine the *adaptability* of the nutrition guidelines. Assessing guideline adaptability was deemed to be the best strategy because it allowed the evaluation to focus on two key aspects of the guidelines; methodological quality and implementability [6]. Assessing and improving guideline adaptability is necessary in order to develop more effective and safer nutritional interventions and guidelines that incorporate best available evidence with evidence-informed implementation techniques.

Three specific evaluation questions were addressed: (1) how adaptable are the guidelines in their current form? (2) what are the key characteristics of the nutrition guideline development process? and (3) how can nutrition guideline development be modified in order to attain more adaptable guidelines?

The evaluation focused on (a) documenting the evidence-informed nutrition guideline development process, (b) identifying where it diverges from the Handbook procedures, and (c) deriving benefits or drawbacks of the nutrition approach. The evaluation followed a rapid assessment procedure structure (RAP) [7]. As such, it started with a pre-established question and conceptual model, while also incorporating RAP specific features such as (a) a focus on a narrow question, (b) small samples of key informants, (c) a short period of field research, (d) interview guides focusing on specific topics, and (e) multiple data collection methods [7]. The study design, data collection, and reporting of the findings were guided by the 11 criteria described by Utarini and colleagues [7]. Information on each criterion is provided in the present section.

The data collection was guided by a fieldwork research guide developed for this evaluation based on experts, staff, and stakeholders’ feedback. The main data sources for this evaluation were official documentation and feedback (both qualitative and quantitative) from the key stakeholders previously involved in the development of nutrition guidelines. The qualitative data was collected through a desk review and semi-structured interviews. Guideline adaptability was assessed quantitatively using two standardized instruments. The *Appraisal Guideline for Research and Evaluation* questionnaire, version II (AGREE II) [8] was used to assess guideline quality, while implementability was assessed with the electronic version of the *GuideLine Implementability Appraisal* (eGlia) [9].

To further strengthen the study findings, the evaluation relied on a mixed methods approach, which included the key features such as rich contextual information and triangulation of data. Different methods were used to address each one of the study research questions. The study methods and research questions are summarized in Table 1.

**Table 1:** Data collection methods

Research question	Data collection	Data analysis
(1) How adaptable are the guidelines in their current form?	AGREE II	Questionnaire results (mean by domains)
	eGlia	Questionnaire results (reconciliation of binary yes/no answers by domain)
	Semi-structured interviews with members of the GDG	Axial coding
(2) What are the key characteristics of the WHO-EPG guideline development process?	Desk review	Axial coding
	Feedback from WHO-EPG experts	Axial coding
	Semi-structured interviews with members of the GDG	Axial coding
(3) What are the recommendations to produce more adaptable guidelines?	Semi-structured interviews with members of the (GDG)	Axial coding

*GDG* guideline development group on nutrition, *EPG* Evidence and Programme Guidance unit, Department of Nutrition for Health and Development

### Desk review and feedback from technical staff

The desk review encompassed peer-reviewed literature on the nutrition evidence-informed guideline development approach [3, 10–12], meeting records of the WHO Guideline Development Groups and Nutrition Guideline Steering Committee (*n* = 3), guideline drafts and external comments on them by external experts responding to the calls for comments (*n* = 6), internal meeting agendas and minutes spanning from 2008 through 2013 (*n* = 14), feedback from key staff from NHD, and a sample of three published evidence-informed nutrition guidelines [13–15] that were checked against the WHO Handbook for guideline development. Key NHD staff (*n* = 3) provided feedback using a questionnaire that covered the main features of each guideline development step, the pros and cons of the process, and existing enabling factors/barriers to guideline adaptability. The three guidelines were assessed using the Handbook as the guide. Departures from the process outlined in the Handbook were documented to identify potential factors that facilitate or impede guideline adaptability. All other data sources were analyzed using axial coding and the same categories used to analyze the interview data, described in detail below. Results from the desk review and the interviews were not pooled together but triangulated, as shown in the results section.

### Assessment of guideline methodological quality and implementability

The guideline adaptability assessment evaluates the methodological quality and implementability of the nutrition guidelines. The adaptability assessment was based on seven guidelines, which were randomly selected from the nutrition guidelines published between 2011–2014 [13–19].

Each guideline was evaluated by one or two external experts who were involved in different capacities with the development of nutrition guidelines. The experts self-selected to participate in the study, which reflects their engagement with the guideline development process and their interest in seeing it improving. Experts were not able to choose guidelines to appraise. The assignment was done by the evaluators, who assured that the experts appraised a guideline with which they had not been directly involved. About half of the appraisers were technical experts and the other half implementation experts. The appraisers were balanced by geographic region and discipline of expertise.

The methodological quality was assessed through the *Appraisal Guideline for Research and Evaluation* questionnaire, version II (AGREE II) [8] instrument. This instrument has 23 items, organized in seven domains: (1) scope and purpose, (2) stakeholder involvement, (3) rigor of development, (4) clarity of presentation, (5) applicability, (6) editorial independence, and (7) overall assessment [8, 20]. Responses are gaged with a 7-point Likert scale and allow open-ended comments for each item. AGREE II has been shown to be valid and easy-to-use [20] and is the most commonly used guideline appraisal instrument [21]. Domain scores are independent and cannot be aggregated to a single score. A quality score is calculated for each domain (this does not apply to the overall assessment domain) by summing up the scores of the individual items within a domain and then scaling the total as a percentage of the maximum possible score for that domain.

- ➢ Maximum possible score = 7 (strongly agree) × 3 (items) × 4 (appraisers) = 84
- ➢ Minimum possible score for domain = 1 (strongly disagree) × 3 (items) × 4 (appraisers) = 12
- ➢ Scaled domain score = [obtained score – minimum possible score]/[maximum possible score – minimum possible score].

The user’s manual does not specify cutoff points, nor does it set minimum domain scores or patterns of scores across domains to differentiate between high-quality and poor-quality guidelines. We were not interested in carrying out a between-guideline comparison, but rather, we wanted to evaluate the quality of the guidelines as a whole. For this reason, the interpretation of the results in this evaluation was done based on the average score obtained by domain across all guidelines.

Implementability was measured by using eGlia, a validated questionnaire for guideline implementability assessment [9]. The eGlia questions are grouped in eight domains: (1) executability, (2) decidability, (3) validity, (4) flexibility, (5) effect on process of care, (6) measurability, (7) novelty/innovation, and (8) computability. In addition, the instrument has a few questions covering “global considerations”. The eGlia questionnaire consists of 30 yes/no items, which are used to evaluate the implementability of the recommendations in a guideline. No scoring is involved in the process of obtaining the results, as Yes/No answers are taken as binary responses. Nevertheless, the eGlia required the evaluators to reconcile the appraisers’ answers; for questions where both “yes” and “no” answers were obtained, the evaluators reconciled the answer as “no”. This conservative interpretation aimed to capture all the potential barriers to implementability. The instrument does not address the implementability of the overall guideline.

The evaluation used the online versions of the AGREE II and eGlia questionnaires [22]. The nine appraisers reviewed a total of thirteen electronic questionnaires (6 AGREE II and 7 eGlia). The respondents’ open-ended comments to AGREE II and eGlia were registered and included in the interpretation of the results.

It is important to consider that neither the AGREE II nor the eGlia questionnaires have been validated for use in the field of nutrition. However, Agree II has demonstrated pertinence [23] and validity [24, 25] in other fields and has been used to assess nutritional interventions [26]. As for the GLIA instrument, there is evidence indicating that it has content and construct validity [27]. Hence, though not evaluated thoroughly for the particular case of nutrition guidelines, these instruments seem to have adequate psychometric properties.

### Semi-structured interviews

A total of twelve semi-structured interviews were conducted for this study across two waves. The interviews explored the key characteristics of the evidence-informed guideline development process, the adaptability of the nutrition guidelines, and suggestions for improvement; dimensions corresponding to the research questions are shown in Table 1. The interviews were conducted in two waves using semi-structured interview guides for each. Questions were designed to gather the informants’ perspectives in a non-leading fashion. A series of follow-up probing questions were also included to explore in detail the ideas that arose during the interviews. Each wave had different participants, and the interviewees were selected through purposive sampling [28]. In our case, we sought to interview individuals who had attended a WHO nutrition guideline development group meeting in the past year. Most interviewees were contacted and interviewed over the course of a guideline development group meeting, the others were referred by NHD staff. Wave 1 was conducted in June 2014 and Wave 2 in October of the same year. Some interviews were conducted face-to-face while others were conducted over the phone or Skype. All interviews were confidential and were audio-recorded. All data management and analysis were conducted using a participant encoded identification number. Interviewees provided verbal consent to be interviewed and recorded and to use the information for the purposes of the evaluation. The audios were transcribed verbatim to be analyzed, all files will be destroyed after 3 years. The first wave (*n* = 8) of semi-structured interviews complemented the data collected from the desk review and questionnaires. Wave 2 (*n* = 4) probed the emerging themes from wave 1 and further asked respondents to formulate recommendations to improve adaptability.

The main selection criterion for the interviewees was heterogeneity; the evaluators actively sought to interview individuals with different areas of expertise and who worked in different WHO regions. Half of the interviewees were technical specialists, and the other half implementation experts; all had substantial experience in guideline development and program implementation. Technical experts were members of the Guideline Development Group “GDG” whose area of expertise is the technical evaluation of the available evidence and can provide feedback on the methodological quality of a guideline. Implementation experts were members of the GDG whose area of expertise is the science of implementation and can provide key insights into guideline feasibility. The sample was comprised of an equal number of males and females. The combined experience of the participants covered the Americas, South-East Asia, Eastern Mediterranean, Western-Pacific, and the European region. All participants had attended at least one GDG meeting in the year previous to the evaluation. The GDG is multidisciplinary and composed of individuals from all WHO regions likely to use the guideline with a membership that is balanced in terms of gender and geography [10]. This group advises WHO on the scope of the guideline, the development of key questions in PICO format, selection of outcomes that guide the evidence reviews and focus the recommendations, use of the Grading of Recommendations Assessment, Development and Evaluation (GRADE) approach for assessing the quality of the evidence, interpretation of the evidence, and the formulation of recommendations [29].

Both waves of interviews were closed after an interim assessment, which indicated that saturation had been reached [30]. The analysis of the semi-structured interviews proceeded through axial coding. Axial coding prioritizes the identification of consistent themes guided by pre-defined categories. Accordingly, a coding framework was developed following a deductive approach. We used pre-defined categories from the study objectives and interview guide. The framework contained all categories and subcategories to be used to tag the interview transcripts. As the coding process continued, the framework was expanded by adding codes to capture concepts that participants brought up in the interviews.

### Data quality control

Various strategies were used to assure the reliability and validity of data. The first was data triangulation, which compared findings from different sources to ensure consistency of results [31]. The results from the desk review and the two waves of interviews were triangulated and indicated convergence, which attests to data quality.

Saturation is present when no new information emerges from additional data [30]. Therefore, saturation is widely used as an indicator of data quality. To assess the level of saturation in interview data, transcripts were first ordered by interview date and then placed into small groups of equal number (wave 1) or assessed one by one (wave 2). For wave 1, each small group was assessed against the previous one for the appearance of new concept codes. For the first wave of interviews, saturation was reached in the second group. In the second wave of interviews, saturation was reached in the third interview, meaning that no new concepts appeared in this transcript, thus suggesting robust data consistency.

Two evaluators, external to WHO, conducted this evaluation. Both had experience in qualitative research in the social and health sciences. This background included a combined experience in other programmatic evaluations as well as knowledge in qualitative interviewing techniques and data analysis. This research project was conducted in compliance with the principles delineated by the United Nations Evaluation Group [32].

## Results: assessing the adaptability of who nutrition guidelines

The main aim of the evaluation was to assess the adaptability of the nutrition guidelines produced by the NHD group at WHO. In this study, adaptability is defined as having two components: methodological quality and implementability [6]. This section draws from the various data collection methods used in this study and presents the results in four subsections: (1) methodological quality, (2) implementability, (3) organizational dynamics, and (4) recommendations to improve guideline adaptability.

### Methodological quality

The guidelines’ methodological quality was assessed using the AGREE-II questionnaire. The results were triangulated with data from the semi-structured interviews and the desk review, which focused on identifying the key characteristics of the guideline development process. Table 2 shows the results of the AGREE-II questionnaire, and Table 3 shows the concepts that emerged in the semi-structured interviews pertaining guideline methodological quality.

**Table 2:** Methodological quality of nutrition guidelines as assessed with Agree II instrument

Agree II Domain	Guideline 1	Guideline 2	Guideline 4	Guideline 6	Guideline 8	Guideline 10	Mean %
Scope and purpose	86	100	100	78	89	86	90
Stakeholder involvement	78	78	50	44	78	72	67
Rigor of development	93	96	98	79	85	77	88
Clarity of presentation	92	94	89	67	83	89	86
Applicability	73	88	54	13	63	65	59
Editorial independence	96	100	100	100	100	100	99

The table shows the quality score of each domain

**Table 3:** Semi-structured interviews: concept frequency for guideline methodological quality

Concept	Wave 1			Wave 2			Overall
Methodological quality	S	W	Total	S	W	Total	
Method to formulate recommendation	4	14	18	5	22	27	45
Systematic review	20	7	27	9	8	17	44
Scope and purpose	12	1	13	4	0	4	17
COI	6	2	8	6	1	7	15
Stakeholder involvement	2	2	4	1	0	1	5

*COI* conflict of interest

**Fig. 1:**
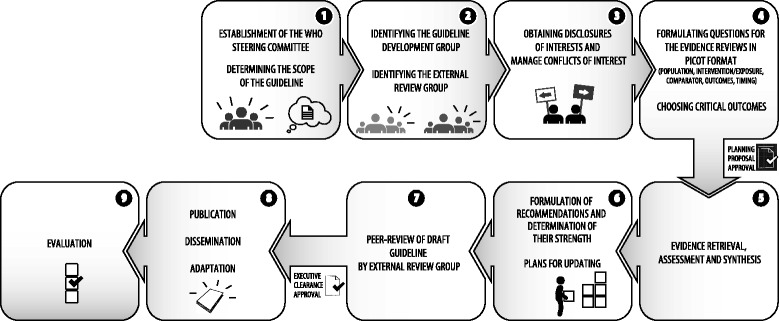
Steps of the WHO guideline development process

The evaluation results indicate that the nutrition guideline development process has improved over the years, resulting in guidelines of higher quality. Guidelines were rated by the study participants as rigorously developed; evidence-based, clearly scoped, and unbiased (Table 2). The AGREE-II indicates that the guidelines perform very well in the domains “scope and purpose,” “rigor of development,” and “editorial independence.” These results are highly congruent with the interview data (Table 3), where interviewees identified the systematic review, the scoping and purpose of the guidelines, and the management of the conflict of interest as methodological strengths. Findings from the desk review further support these findings, by suggesting good performance in the same areas and showing that the Department of Nutrition for Health and Development follows closely the guideline development process described in the Handbook [4, 5] (shown in Fig. 1).

One area where WHO nutrition technical staff performs exceptionally well is in the management of conflicts of interest (COI) (step 3) (AGREE-II domain; editorial independence), which assures transparency in the conduct of the meetings and the decision-making process for developing recommendations. This happens through careful screening of the previous publications and projects of prospective GDG members, an analysis of potential real or perceived conflicts of interest, and declaration of interests (financial and non-financial) by all external contributors to guideline development. Individuals who declare one or more relevant interests that are deemed relatively minor in significance may be allowed to participate in the meetings because of their expertise; however, they are asked to not participate in deliberations or even to be present in closed sessions of the meeting when decisions are being made on recommendations related to their declared interests. An adequate management of the COI is paramount for guideline quality, as it assures that the guideline development is independent from its funding bodies [33, 34].

A second area of strong performance is the evidence retrieval, summary and assessment, and systematic reviews (AGREE-II domain; rigor of development) (step 5). The methods to search, select, and assess the evidence are clearly described. Further, the Department of Nutrition for Health and Development has built capacity to produce high-quality systematic reviews when a high-quality summary of the evidence is not available. More recently, the Department has requested that ongoing and unpublished trials are documented as part of the systematic process [35], through the search of the International Clinical Trials Registry Platform (CITRA) hosted at WHO and by contacting relevant organizations. These efforts have resulted in a partnership with the Cochrane group and the development of a Cochrane field focused on nutrition.

Nonetheless, the desk review and interviews indicated that the step involving the formulation of recommendations (step 6) can be improved (AGREE-II; rigor of development). This pertains specifically to the process used by the GDG to establish the strength of the recommendation. The strength of a recommendation conveys the degree to which the GDG is confident in the balance between the desirable and undesirable consequences of implementing a recommendation [5]. A recommendation can be strong or conditional. Our evaluation’s results indicate that the conditional category is currently being underused. For example, the semi-structured interviews showed that the GDG tends to be reluctant to use the “conditional” category to rate the strength of the recommendations they issue. Further, when a recommendation falls under this category, the group needs to communicate more clearly the level of uncertainty implied in the conditional category, so guideline users can implement the recommendation accordingly. In addition, gray literature on values and preferences of the populations affected by the recommendation tends to be overlooked. This is problematic because population values and preferences reveal the importance people assign to intervention effects and because values and preferences are supposed to be taken into account when deciding the e strength of any recommendation [5]. This problem of not including values and preferences was also made evident in the interviews (concept; method to formulate recommendation) and was further reflected by a low score in the AGREE-II stakeholder involvement domain that assesses, among other things, the degree to which the patients’ views and preferences have been sought.

Lastly, the scoping of the question and purpose of the guidelines (AGREE-II domain) (step 4) is an area of good performance. The guidelines’ objectives and research questions were considered adequate and unambiguous. In addition, the population to whom the guideline applies is adequately specified.

### Guideline implementability

Guideline implementability was assessed through the eGlia questionnaire. The results of this assessment are shown in Table 4 (definitions of each one of the eGlia domains can be found in Appendix). Further insights into the topic of guideline implementability were drawn from the semi-structured interviews, the AGREE-II (applicability domain), and the desk review. Table 5 shows the concepts pertaining guideline implementability that emerged during the interviews. The frequency of each one of these concepts provides a sense of the relative importance of each concept.

**Table 4:** eGlia results by domain

Implementability domain/recommendation	1	2	3	4	6	8	10a	10b
Global assessment	✓	✓	No	No	No	No	No	No
Executability	✓	✓	No	No	✓	No	✓	No
Decidability	✓	✓	No	✓	No	✓	✓	No
Validity	✓	✓	✓	✓	✓	✓	No	No
Flexibility	✓	✓	✓	✓	No	No	No	No
Effect on process of care	No	✓	No	✓	✓	✓	No	No
Measurability	No	✓	No	✓	No	✓	✓	✓
Novelty/innovation	No	✓	No	✓	✓	No	No	No
Computability	✓	✓	✓	✓	✓	✓	✓	No

“No” indicates that a recommendation does not meet the criterion of implementability in the specific domain

eGlia addresses the implementability of the recommendation. It does not address the implementability of the guideline as a whole. For example, guideline 10 has two recommendations

**Table 5:** Semi-structured interviews: concept frequency for guideline implementability

Concept	Wave 1			Wave 2			Overall
Implementability	S	W	Total	S	W	Total	
Applicability	1	21	22	0	6	6	28
Executability	0	9	9	0	6	6	15
Validity	0	5	5	0	1	1	6
Relevance	6	0	6				6
Impact	0	6	6				6
Decidability	0	4	4				4
Novelty	0	2	2				2
Measurability	0	1	1	0	1	1	2
Problem-based guidelines	0	1	1				1
Flexibility^a^				0	1	1	1
Cost^a^				0	1	1	1

*S* strength, *W* weakness

^a^Concept emerged in wave 2

Indications about the need to improve guideline implementability were a consistent finding throughout the data sources used in this study. Notably, the interviewees clearly and repeatedly identified the applicability and executability of the guidelines to be a weakness (eGlia; executability) (interview concepts; applicability and executability). First, there is a need for specific strategies and tools to implement the guideline’s recommendations. Second, recommendations need to state more explicitly the actions to be taken. While there are different perspectives within WHO on how directive the guidelines should be, there was ample consensus in that guidelines need to assess implementation barriers and consider costs, resources, and potential impact on health systems (eGlia, global assessment; AGREE-II, applicability; and interviews). Results also indicate that the guidelines do not permit much interpretation and that they don’t allow for alternatives in execution, which is a barrier to implementation. Lastly, the recommendations tend to require novel and unconventional behaviors for clinicians or patients, which further challenges their implementability (eGlia; novelty/innovation).

Lastly, the desk review indicated that the nutrition guideline development process should improve the dissemination, adaptation, and implementation of the nutrition guidelines (step 8) (AGREE-II; applicability). These limitations had been informally captured by the technical staff, and more focus has been given to these considerations in the past few years. Lastly, the desk review indicated that the department of Nutrition for Health and Development needs to put in place a strategy to evaluate the impact of the guidelines (step 9), as it lacked one at the time of this evaluation.

#### Organizational dynamics

The semi-structured interviews produced valuable information on specific aspects of the guideline development process that impact guideline adaptability and that were not in the initial framework of the evaluation. The interview results are shown in Table 6.

**Table 6:** Semi-structured interviews: concept frequency for organizational dynamics

Concept	Wave 1			Wave 2			Overall
Organizational dynamics	S	W	Total	S	W	Total	
Group diversity	3	10	13	14	13	27	40
Decision-making process	4	12	16	2	12	14	30
Meeting	1	16	17	1	2	3	20
Departures from standard practice	1	0	1	5	0	5	6
Guideline prioritization^a^				0	6	6	6
Speed of process^a^				0	6	6	6
Problem vs. Intervention^a^				0	5	5	5
Steps	4 (descriptive)		4				4
WHO features	3 (descriptive)		3				3
Update	0	3	3				3

*S* strength, *W* weakness

^a^Concept emerged in Wave 2

This emerging information allowed the identification of three aspects of the guideline development process that merit improvement. These pertain group dynamics operating in the GDG that are shaped to some extent by the institutional framework provided by the Nutrition for Health and Development group. Because these pertain issues resulting from interactions, it comes to no surprise that these aspects only emerged in the interviews. These are the management of the *group diversity*, the *decision-making process*, and the group *meetings*. These issues deserve attention for their potential to negatively impact guideline adaptability. The *diversity* of the GDG members in terms of expertise potentially enables debates and the integration of varied ideas and perspectives. Yet, academic knowledge tends to be privileged over implementation knowledge; thus creating a dynamic where the implementers refrain from debating, thereby undermining the very purpose of their participation in the GDG. Thus, it is necessary to devise strategies to integrate the diversity in knowledge that the GDG members can provide.

The *decision-making process* was described as straightforward and successful in fostering evidence-informed recommendations. However, it is negatively affected by the way evidence is presented at the GDG *meetings*. In most cases, no information is sent prior to the meeting, which requires participants to ponder considerable amounts of information on the spot. The very full agenda limits the time available to review large volumes of complex evidence. More recently, at guideline development group meetings, WHO nutrition technical staff have started to present the evidence with a full description of the reasons for excluding specific studies. This helps achieve transparency and avoids distractions from discussions of potentially missed studies at meetings where time is critical.

### Recommendations to improve the adaptability of WHO nutrition guidelines

One of the aims of the evaluation was to elicit recommendations coming from the study participants to understand how guideline adaptability can be improved. The present evaluation collected a sizeable number of recommendations through the semi-structured interviews. The recommendations pertained the same areas discussed above and focused on “methodological quality,” “organizational dynamics,” and “implementability.” The recommendations are presented in Table 7 and summarized below.

**Table 7:** Semi-structured interviews: recommendations for the improvement of guideline adaptability by category and concept frequency

Recommmendations	W1	W2	Total
Implementability	116	136	252
In or out	30	23	53
Package	14	38	52
Implementation guidelines	17	31	48
Applicability	8	13	21
Higher relevance	3	18	21
Improved usefulness	12	6	18
Executability	9	5	14
Measurability	12	1	13
Cost	7	1	8
Decidability	2		2
Impact	1		1
Novelty	1		1
Organizational dynamics	23	53	76
Decision-making process	13	28	41
Meeting	4	13	17
Group diversity	5	11	16
Update	1		1
Guideline prioritization		1	1
Quality of guidelines	24	19	43
Method to formulate recommendation	11	11	22
Stakeholder involvement	6	5	11
Systematic review	5	3	8
COI	2		2
Scope and purpose	0		0
Overall	163	208	371

The topic of implementability elicited the largest number of recommendations (*n* = 252, Table 7). In order to overcome an overly narrow guideline focus, respondents recommended developing guideline “packages.” These would address wider nutrition relevant public health problems (“package” code in Table 7) and would be developed in collaboration with policymakers, who could identify the challenges of combining or integrating various interventions. While the development of these “packages” brings about new challenges (e.g., lack of evidence on how to best combine interventions; trade-offs between intervention depth and breadth), participants considered that the benefits are likely to outweigh the costs.

All participants agreed that explicit and precise implementation advice is highly valuable but largely missing from nutrition guidelines. Two possible solutions were proposed: developing separate implementation guidelines or providing implementation guidance within the current guidelines (“in or out” concept Table 7). The biggest challenge to the first option is the scarcity of studies that could inform the development of evidence-informed implementation guidelines. Nonetheless, WHO could consider including more implementation considerations within the current guidelines. This would entail describing strategies to promote guideline use at the local level and discussing interventions to overcome implementation barriers (contextual, monetary, etc.). Further guidance could also discuss the novelty of the proposed recommendations, so potential barriers to implementation can be identified in a timely manner. Because this information is more likely to appear in gray literature and highly contextualized studies (mostly employing qualitative methods), WHO could rely on the Cochrane guidelines for systematizing qualitative evidence to produce and provide nutrition implementation advice and could also focus on requesting feedback from guideline users.

The semi-structured interviews also produced a sizeable number of recommendations to improve the organizational dynamics. A first set of recommendations (*n* = 76, Table 7) focuses on group diversity and decision-making. The diversity in expertise within the GDG is both a big advantage and a challenge. Interviewees advised promoting a productive dialog between GDG technical and implementation experts. These value each other’s expertise, but they have not yet started working in an integrated way. WHO can take action by establishing a clear standpoint about the role of implementers and by establishing clear expectations for both groups. In addition to the current training on epidemiological methods, WHO could also train GDG members on guideline implementability. Further, the chair and vice-chair at GDG meetings should be individuals with a strong background in both areas, so they can ensure that all these issues are discussed in-depth.

To improve decision-making, the interviewees recommended that the WHO group for nutrition for health and development group presents evidence more concisely and in a timely manner. The information could be shared before the GDG meeting. This will allow avoiding the oversaturation with information and allow sufficient time for GDG members to process all relevant information.

The interviews produced useful recommendations for improving guideline methodological quality (*n* = 43, Table 7). These focused mostly on the mechanisms to determine recommendation strength. First, the approach outlined in the WHO Handbook should be more closely followed when deciding on the strength of a recommendation. More guidance is needed during the GDG meetings on how to determine the strength of recommendations [36–39]. This would likely result in more “conditional” recommendations [5]. Recently, the GDG in nutrition has established decision rules for the GDG. A recommendation can be finalized either by unanimous decision (the primary decision rule) or by a secondary decision rule, which requires an agreement of 2/3 of those allowed to participate in decision-making (i.e., those who have been assessed as able to participate in after the management of any conflicts of interest). This change has greatly improved and facilitated the process of determining the strength of the recommendation.

Values and preferences of persons affected by the recommendations need to be considered when formulating recommendations. This requires drawing from observational and qualitative evidence and using primary data collection methods. Also, practical considerations such as cost, resources, and impact on the health system need to be accounted for. Interviewees recommended that WHO technical staff in nutrition prepare this information and present it at the GDG meetings.

## Discussion

The current evaluation focused on the guideline development process at WHO led by the Evidence and Programme, Guidance, Department of Nutrition of Health and Development, and used a mixed methods approach to (1) determine the level of adaptability of its guidelines (i.e., methodological quality and implementability), (2) identify the key characteristics of the evidence-informed guideline development process, and (3) produce recommendations to improve future guideline development in nutrition actions for health and development. The most salient strengths of the current process and methods are the management of conflicts of interest and the systematic use of high-quality evidence to inform the recommendations. The key areas for improvement are the limited implementability of recommendations and the challenges related to collaborative work within interdisciplinary groups.

Recent research indicates that some of the difficulties identified in this evaluation, specifically, those pertaining the determination of the recommendation strength and the decision-making process, may be partially due to limitations of the GRADE guidance itself when assessing the quality of the evidence. For instance, the decision-making process can be hindered because the GRADE guidance has too few categories to capture different levels of evidence other than randomized controlled trials and because it is highly time-consuming, which may thus not make it suitable for a grading of evidence under time pressure [40]. Nonetheless, individual factors also contribute to the identified problems. Research indicates that strong recommendations may be issued based on weak evidence because of political considerations, pre-conceptions about the benefits of a recommendation regardless of the evidence, and concerns that conditional recommendations will be ignored [41]. In this sense, guideline adaptability can be improved not only by addressing the individual level factors but also by seeking innovative solutions to problems that are intrinsic to the methodologies that are currently at use.

To continue improving the process, WHO needs to conduct periodic evaluations and act on results. Notably, the Department of Nutrition for Health and Development has started to implement some of the recommendations formulated in this evaluation. The latest guidelines have started to provide implementation guidance in the form of considerations related to equity, implementation, ethics, and regulatory aspects. While this remains an important step in the right direction, a recent study of the implementation guidance provided by the WHO guidelines shows an institutional tendency to emphasize policy-based implementation techniques as opposed to evidence-based active techniques, which demonstrates the need to build stronger guidance for implementation into the guidelines. Active implementation techniques include follow-up and personal interaction with the implementers, audit and feedback, educational outreach, and use of opinion leaders [42]. Recent guidelines have a section on the efficacy and the effectiveness of proposed interventions [43]. Such information complements the strategies provided in the WHO e-Library of Evidence for Nutrition Actions (eLENA) [44].

Another recent change is the ongoing development of guideline packages to be published by mid 2017 (i.e., nutritional anemias prevention and control; a practical handbook for health care workers). With this change, WHO expects to increase the relevance of the nutrition guidelines because these will now fit better into the dynamics of health systems, as decision makers generally address broad problems such as “nutrition in antenatal care” or “anemia” or focus on target populations such as “infants 6–23 months of age.”

The Handbook [4, 5] recommends conducting qualitative and quantitative studies to assess intervention effectiveness and its determinants, including social determinants of health, contextual barriers, and facilitators, as well as values and preferences of the target population. Through the collection of such evidence, WHO can identify which tools and strategies work across contexts. To this end, WHO can use the methods proposed by the Cochrane Qualitative and Implementation Methods Group [45, 46]. Data should be collected from the early stages of implementation, to confirm the baseline for subsequent evaluations. In addition, the number of countries that has implemented the guideline should be monitored as a useful indicator of guideline uptake. Pilot and prospective studies to assess the intervention effects are needed. The group on nutrition for health and development may also want to conduct further studies to identify the determinants of successful implementation.

Changes are currently underway to improve other issues identified in this evaluation. Among these, WHO has started to monitor guideline implementation in different countries. This is a positive step, as this will generate evidence on what works and under which circumstances [5] which can inform future guidelines, creating the right conditions to improve the implementability of future guidelines.

The present evaluation also has limitations. It drew primarily from individuals who have collaborated with WHO to develop evidence-informed guidelines on micronutrients and nutrition actions; the data could therefore be biased as a result of social desirability (respondents may be reluctant to provide answers seen as undesirable). To help to overcome such potential biases, anonymity was assured, allowing frank and in-depth discussions of the guideline development process. Further, the qualitative approach of this project benefits from collecting information from the key informants, who are highly engaged in guideline development and who can provide a critical assessment of its strengths and problems. To further strengthen data validity and credibility, the information was triangulated from different sources.

Commissioned by the unit of Evidence and Programme Guidance, Department of Nutrition for Health and Development, the present evaluation identified approaches to improve the adaptability (i.e., methodological quality and implementability) of future guidelines. The evaluation also identified strengths and weaknesses, as well as the catalyzers and barriers of its current guideline development process.

## Conclusions

This study focuses on the guideline development process at WHO led by the Evidence and Programme, Guidance, at the NHD department. Our research shows that the current process has several strengths, among them are the appropriate management of conflicts of interest and the systematic use of high-quality evidence to inform the recommendations. The key areas for improvement are the limited implementability of recommendations, the lack of explicit and precise implementation advice in the guidelines, and challenges related to collaborative work within interdisciplinary groups. As WHO relies heavily on a standardized procedure to develop guidelines, the lessons learned may be applicable to guideline development across the Organization and to other groups developing guidelines.

## Appendix

Guideline implementability: eGlia

Developed at Yale University in 2005, eGlia is the only validated questionnaire to assess guideline implementability. The eGlia domains gage whether the guideline addresses the following dimensions:

- Global considerations
- Executability: whether the guideline provides actions to be taken.
- Decidability: conditions (e.g., age, gender, clinical findings, and laboratory results) under which actions should be taken.
- Validity: degree to which the recommendation reflects the intent of the developer and strength of evidence.
- Flexibility: degree to which a recommendation permits interpretation and allows for alternatives in its execution.
- Effect on process of care: degree to which the recommendation impacts the usual workflow in healthcare facilities.
- Measurability: degree to which the guideline identifies markers to track the effects on implementation.
- Novelty/innovation: degree to which the recommendation proposes behaviors considered unconventional by clinicians or patients.
- Computability: ease with which a recommendation can be operationalized in an electronic information system (only applicable when an electronic implementation is planned).

## Abbreviations

Agree II: The Appraisal Guideline for Research and Evaluation questionnaire, version II
eGlia: Guideline Implementability Appraisal, electronic version
eLENA: WHO e-Library of Evidence for Nutrition Actions
GDG: Guideline Development Group
GRADE: Grading of Recommendations Assessment, Development and Evaluation
NHD: Evidence and Programme Guidance, at the Department of Nutrition for Health and Development
RAP: Rapid assessment procedure structure
WHO: World Health Organization
